# Analysis of Spinopelvic Sagittal Balance and Persistent Low Back Pain (PLBP) for Degenerative Spondylolisthesis (DS) following Posterior Lumbar Interbody Fusion (PLIF)

**DOI:** 10.1155/2020/5971937

**Published:** 2020-01-11

**Authors:** Shuangjun He, Yijian Zhang, Wei Ji, Hao Liu, Fan He, Angela Chen, Huilin Yang, Bin Pi

**Affiliations:** ^1^Department of Orthopedics, The People's Hospital of Danyang, Danyang 212300, China; ^2^Department of Orthopedics, The First Affiliated Hospital of Soochow University, Suzhou 215006, China; ^3^University of Waterloo, Waterloo, Canada

## Abstract

**Objective:**

To investigate the change of spinopelvic sagittal balance and clinical outcomes after posterior lumbar interbody fusion (PLIF) in patients with degenerative spondylolisthesis (DS), especially the relationship between sagittal spinopelvic parameters and persistent low back pain (PLBP).

**Methods:**

107 patients who were diagnosed with DS and underwent PLIF in our department were enrolled retrospectively in the present study. Sagittal spinopelvic parameters including lumbar lordosis (LL), segmental lordosis (SL), height of the disc (HOD), sacral slope (SS), pelvic incidence (PI), and pelvic tilt (PT) were recorded pre- and postoperatively. Sagittal balance and clinical outcomes were compared between patients with and without PLBP. Pearson correlation was used to analyze the change of sagittal balance parameters and clinical functions. Logistic regression analysis was performed to examine the risk factors of PLBP.

**Results:**

It showed significant improvements of SL, HOD, and PT postoperatively. Both the Numeric Rating Scale (NRS) and Oswestry Disability Index (ODI) had significant improvement postoperatively. Change of PT and SL also differed observably between patients with and without PLBP. SL and PT were correlated with NRS and ODI, and insufficient restoration of PT was an independent factor for PLBP.

**Conclusion:**

The sagittal balance parameters and clinical outcomes can be improved markedly via PLIF for treating DS. Restoration of SL and PT was correlated with satisfactory outcomes, and adequate improvement of PT may have positive impact on reducing PLBP.

## 1. Introduction

Degenerative spondylolisthesis (DS) is a common lumbar degenerative disease that involves mechanical low backache, radiculopathy, and neurologic claudication [1]. In addition to conservative treatments including nonsteroidal anti-inflammatory drugs (NSAIDs), traction treatment, and extensor exercises, surgical treatments including posterior approach fusion: PLIF, posterolateral approach fusion: PLF, anterior approach fusion: ALIF, and extreme lateral approach fusion: XLIF were recommended for these patients [2]. To date, PLIF is considered to be the optimal procedure due to its high fusion rate and effective decompression of neural roots [3]. However, some patients complain about new onset or persistent low back pain after PLIF surgery, known as “persistent low back pain” (PLBP) or “failed back surgery syndrome” (FBSS) [4]. Unfortunately, there is for sure no one exact cause for PLBP. It was reported that degeneration of paraspinal muscle may be the risk factor for postoperative PLBP [5]. Meanwhile, excessive damage of multifidus muscle, during surgery was considered, may result in the occurrence of PLBP [6].

Recently, restoration and improvement of spinopelvic sagittal balance has gained much attention in lumbar fusion surgery. Previous studies reported significantly larger PI [7] and smaller LL in symptomatic patients compared with normal controls [8]. A negative correlation between PT and PLBP in spondylolisthesis patients has also been found, indicating that lumbar degeneration accompanies sagittal balance deterioration [9]. For most DS patients, PLIF can dramatically improve sagittal balance via the fixation of pedicle screws and distraction of the inserted cage [10]. However, few studies have been published about correlation between spinopelvic sagittal balance and clinical outcomes, especially the impact of spinopelvic sagittal balance on PLBP.

In present study, the authors examined the change of spinopelvic sagittal balance in patients undergoing PLIF for DS to investigate the correlation between restoration of spinopelvic sagittal balance and improvement of clinical outcomes. Additionally, the study is designed to verify whether an improvement of sagittal balance had an alleviated effect on postoperative PLBP.

## 2. Patients and Methods

### 2.1. Study Population

We retrospectively enrolled 120 patients treated with PLIF for DS between January 2014 and December 2015 at our department in the present study. 10 patients who did not complete the follow-up and 3 patients who were missing information were excluded. The remaining 107 patients, which consisted of 35 males and 72 females were examined for radiographic and clinical data pre- and postoperatively. The inclusion criteria were as follows: (1) patients with definite diagnosis of DS; (2) patients complained about neural symptoms due to compression; and (3) compression was evident on CT or MRI. The exclusion criteria included (1) multilevel (≥3 levels) lumbar fusion; (2) severe systemic disease; (3) vertebral fracture; and (4) tumors. All patients provided their written informed consent. In addition, the study has been reported in line with the STROCSS criteria.

### 2.2. Surgical Procedure

All patients underwent PLIF procedures as described by Tsutsumimoto et al. [11]. All patients were placed in the prone position, and general anesthesia was used. After routine skin disinfection, a median incision of 6∼8 cm was taken with surgical segment as the center. Then, subcutaneous tissue, muscle, and fascia were separated, and two or four screws (Medtronic, USA) were inserted into the pedicle. Fluoroscopy with a C-arm was performed to confirm the position of the inserted screw. After that, one or two cage (Medtronic, USA) was placed into the intervertebral space following the decompression of nerve roots and removal of the herniated disc, flavum ligament, and articular processes. Finally, fluoroscopy was performed again to check the position of the inserted cage and sutured the incision layer by layer.

### 2.3. Spinopelvic Sagittal Balance

**Figure 1:**
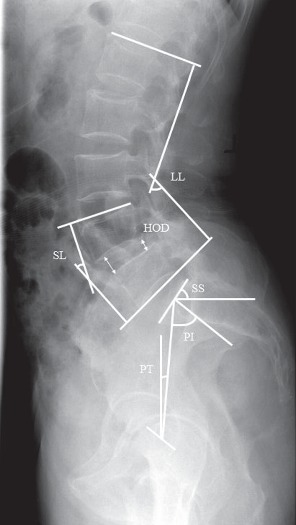
Measurement of spinopelvic sagittal balance (lumbar lordosis, segmental lordosis, sacral slope, height of the intervertebral disc, pelvic incidence, and pelvic tilt) on a lateral radiograph.

In our study, each patient had lumbar spine radiographs with anterior-posterior and lateral position before and after surgery. The specific measurement of sagittal balance of spinopelvic parameters included: LL, SL, SS, HOD, PI, and PT [12, 13] (Figure 1).

### 2.4. Clinical Outcomes

All patients completed NRS and ODI questionnaires to evaluate the improvement of functional scores [14]. The Macnab criteria were also used to evaluate the outcomes, consisting of four levels of clinical efficacy. Patients categorized as III and IV grade or with an NRS reduction of less than 50% last over 6 months were considered as PLBP. Bony fusion criteria were the formation of continuous bone bridge between adjacent vertebrae in X-rays, or without an obvious clear zone between adjacent vertebrae via CT, or without range of motion at the surgical level on dynamic radiographs [15].

### 2.5. Statistical Analysis

**Figure 2:**
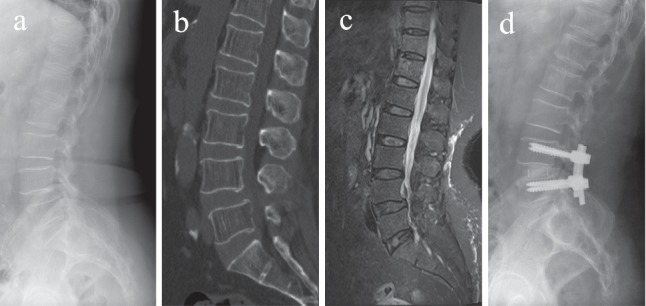
Preoperative sagittal lateral view (a): sagittal computed tomographic scan (b); sagittal T2-weighted magnetic resonance image (c); postoperative sagittal lateral view (d) of a 77-year-old female patient who suffered from degenerative spondylolisthesis at level L4 and underwent posterior lumbar interbody fusion.

SPSS 17.0 (Chicago, IL, USA) was used to collect and analyze the data in this study. Continuous data were showed as mean and standard deviation. Independent *t*-test was used to assess the difference between continuous data, while the Chi-squared test was used to analyze the difference between categorical data. Pearson's correlation coefficient was performed to evaluate the correlation between spinopelvic sagittal balance parameters and clinical outcomes. The logistic regression model was used to assess the influence of each variable on PLBP. *p* values less than 0.05 were considered statistically significant (Figure 2).

## 3. Results

In the present study, 107 DS cases (35 males and 72 females) with the average age 58.5 received PLIF and completed a mean of 18.3 months follow-up of at least one year. The surgical levels were 79 cases of single segment and 28 cases of two segments. Blood loss and operative time were 358.9 ± 61.5 (ml) and 136.4 ± 21.7 (mins), respectively. 95/107 cases (88.8%) achieved bony fusion at the final follow-up (Table 1). No severe intra- or perioperative complications were detected in any of the patients.

Of the sagittal balance parameters, SL (improved from 16.8 ± 7.4 to 20.1 ± 7.1, *p*=0.001), HOD (improved from 8.0 ± 2.6 to 10.3 ± 2.7, *p* < 0.001), and PT (improved from 18.7 ± 4.6 to 15.9 ± 5.7, *p* < 0.001) improved significantly after surgery. In clinical outcomes, NRS (improved from 7.7 ± 0.9 to 3.0 ± 0.9, *p* < 0.001) and ODI (improved from 55.0 ± 6.0 to 23.6 ± 6.7, *p* < 0.001) both improved significantly after surgery (Table 2).

### 3.1. Subgroup Analysis

At the final follow-up, 17 patients who were categorized as fair, poor Macnab criteria, and had a less than 50% reduction of NRS for at least 6 months were considered to have PLBP. In comparison with non-PLBP patients, PLBP patients had significant worse improvement of SL and PT after PLIF. Both recovery of NRS and ODI were dramatically worse in PLBP patients (Table 3).

From the correlation analysis, there was significant correlation between following alteration of sagittal balance parameters: △LL and △SL, △LL and △SS, △LL and △PI, △LL and △PT, △SL and △SS, △SL and △PT, △SS and △PI, and △SS and △PT. Improvement of NRS was correlated with △SL and △PT. Similarly, improvement of ODI was correlated with △SL and △PT (Table 4).

In the logistic regression model, sex, age, operative levels, and most sagittal balance parameters did not have a significant impact on postoperative PLBP. Interestingly, △PT was proved as an independent risk factor of postoperative PLBP (Table 5).

## 4. Discussion

As a most applied surgical approach, PLIF is considered superior to other fusion procedures in terms of anterior column support, sufficient decompression of nerve roots, and restoration of lumbar alignment [16]. Several prior studies have reported the effective improvement of clinical efficacy of PLIF for DS [17]. In corroboration with previous results, both NRS and ODI improved significantly in our study after PLIF. Although most patients can recover satisfactorily after surgery, some patients may complain of prolonged PLBP or FBSS. In the present study, 17 patients (17/107, 15.9%) reported having PLBP. None of the patients received reoperation, and 10 patients recovered gradually through extensor exercises, and 7 patients used NSAIDs to control symptoms. The reasons for PLBP after lumbar surgery have been investigated for decades. It was reported that preoperative long-term backache is a risk factor for postoperative PLBP, and that a higher rate of fatty infiltration can often be detected in PLBP patients [5]. It was also suggested that inadequate decompression, vertebral instability, misjudgement of the responsible level, and recurrent disc herniation are all risk factors for PLBP [18]. Unfortunately, the exact cause of PLBP is still unspecified.

Sagittal balance of spinopelvic has gained much interest recently for its critical role in maintaining the curvature of the entire spine. A previous study indicated that LL, PI, and PT are all closely correlated with sagittal balance, and that PI is a key parameter that can be calculated by the sum of PT and SS [19]. In this study, LL, SL, SS, HOD, PI, and PT were recorded pre- and postoperatively to evaluate spinopelvic sagittal balance. Imbalance of the lumbar and pelvis may be a potential cause for degenerative lumbar disease. Ferrero et al. conducted a study of 654 patients and found that DS is relevant to a large PI and small LL [7]. This result was also found in another study [20], which indicated that imbalanced sagittal parameters are formed gradually during the process of lumbar degeneration. In this study, SL, HOD, and PT all improved dramatically after surgery, indicating the marked positive impact of PLIF on the spinopelvic sagittal balance. Previous studies have demonstrated that deterioration of natural sagittal balance may be correlated with poor clinical outcomes. It was also reported that patients having low back pain showed lower SS and LL compared with normal controls [21]. However, whether sagittal balance restoration can influence clinical outcomes remains controversial [22, 23].

In this study, we conducted the subgroup comparison of PLBP and non-PLBP patients, as well as correlation analysis between spinopelvic sagittal balance and clinical outcomes. We observed three important results. First, compared with the non-PLBP group, the PLBP group had worse clinical outcomes and less improvement of SL and PT, indicating inadequate correction of sagittal balance parameters may be a risk factor for postoperative PLBP. Second, there was noticeable correlation between changes of each sagittal balance parameters, indicating the change of one sagittal balance can be affected by the other. This result also revealed the impact of PLIF on sagittal balance is not alteration of one parameter but two or more parameters. Third, improvement of NRS and ODI also correlated the change of SL and PT, which meant that restoration of sagittal balance may have a positive effect on clinical outcomes. For SL, previous literature had demonstrated that improvement of SL is necessary for favorable clinical functions, and several methods had been proposed to achieve this goal. Melikian et al. suggested that a nearly 30-degree cage insertion combined with the addition of anterior longitudinal ligament release could achieve satisfactory lordosis and did not exert oversized pressure on the vertebral endplate and body [24]. Rice et al. also recommended a curved graft may be superior to obtain a more anatomic structure in lumbar fusion surgery [25]. Based on our experience, we suggested that a moderate-sized cage, proper resection of posterior elements, and anterior placement of cage within disc space may be beneficial for a better SL.

With regard to PT, it is reported that patients with DS have a high PI and PT, perhaps as compensation for pelvic retroversion [26]. Due to its anatomical features, PI is hardly affected by operation since the restoration of PT plays a crucial role in improving sagittal balance. Compared with non-PLBP patients, PLBP patients had significant worse improvement of PT. Furthermore, in logistic regression analysis, PT was found as an independent risk factor for postoperative PLBP. Therefore, we speculate that restoration of sagittal balance, especially the PT value, may play a pivotal role in reducing the occurrence of PLBP. It is considered that sagittal imbalance is an interactive phenomenon that is accompanied with alteration of LL, SS, PI and PT [27]. For DS, insufficient restoration of sagittal balance especially the PT value may result in higher energy expenditure for maintaining compensatory mechanisms subsequently cause the occurrence of PLBP [28]. In contrast, restoration of PT value can improve the range of hip extension and alleviate the need for compensatory mechanisms for an upright posture [29, 30]. According to practice in our team, we considered that a suitable position of the inserted cage is not only beneficial to SL but also conducive to PT. Moreover, long-term and effective exercise of extensors is also important, which can increase the stability of lumbar and help with the PT improvement as the pelvis rotates forward.

Our study had some limitations. Firstly, the sample size of enrolled subjects is not big enough which may introduce some bias. Second, this was a retrospective study and did not use randomization. Third, the follow-up of this study is short; hence, long-term results still need to be studied. Additionally, many patients did not complete the final follow-up, resulting in a lack of fusion data. Lastly, the specific cause of postoperative PLBP is still unclear; a large, long-term, and prospective cohort study is needed to confirm our predictions.

## 5. Conclusion

This preliminary study demonstrated that PLIF can improve spinopelvic sagittal balance and clinical outcomes for DS. Restoration of SL and PT was correlated with better clinical outcomes. A sufficient improvement of PT may have a positive impact on reducing postoperative PLBP.

## Figures and Tables

**Table 1:** Demographic data of patients.

Variables	Data
Number of patients	107
Sex (male/female)	35/72
Age	58.5 ± 11.4
BMI (kg/m^2^)	23.5 ± 2.5
Surgical levels (one/two)	79/28
Blood loss (ml)	358.9 ± 61.5
Operative time (mins)	136.4 ± 21.7
Hospital stay (days)	12.4 ± 1.7
Follow-up (months)	18.3 ± 2.4
Fusion rate	88.8% (95/107)

Abbreviations: LL, lumbar lordosis; SL, segmental lordosis; SS, sacral slope; HOD, height of the intervertebral disc; PI, pelvic incidence; PT, pelvic tilt. ^*∗*^*p* values in boldface indicate statistical significance (*p* < 0.05).

**Table 2:** Change of spinopelvic sagittal balance and clinical outcomes after surgery.

Variables	Preoperative	Postoperative	*p* value
LL	40.4 ± 13.6	43.8 ± 10.8	0.05
SL	16.8 ± 7.4	20.1 ± 7.1	**0.001** ^*∗*^
SS	33.2 ± 11.0	34.8 ± 9.0	0.24
HOD	8.0 ± 2.6	10.3 ± 2.7	**<0.001** ^*∗*^
PI	50.4 ± 10.3	49.9 ± 8.9	0.71
PT	18.7 ± 4.6	15.9 ± 5.7	**<0.001** ^*∗*^
NRS	7.7 ± 0.9	3.0 ± 0.9	**<0.001** ^*∗*^
ODI	55.0 ± 6.0	23.6 ± 6.7	**<0.001** ^*∗*^

Abbreviations: LL, lumbar lordosis; SL, segmental lordosis; SS, sacral slope; HOD, height of the intervertebral disc; PI, pelvic incidence; PT, pelvic tilt; NRS, Numeric Rating Scale; ODI, Oswestry Disability Index. ^*∗*^*p* values in boldface indicate statistical significance (*p* < 0.05).

**Table 3:** Comparison of variables between two groups.

Variables	Patients with PLBP (*n* = 17)	Patients without PLBP (*n* = 90)	*p* value
Sex (male/female)	7/10	28/62	0.42
Age	61.2 ± 12.6	58.0 ± 11.2	0.29
Surgical levels (one/two)	13/4	66/24	1.00
Pre-LL	40.5 ± 10.9	40.4 ± 14.2	0.96
Post-LL	42.2 ± 9.6	44.0 ± 11.0	0.53
△LL	1.7 ± 10.1	3.7 ± 9.1	0.42
Pre-SL	18.6 ± 4.2	16.5 ± 7.8	0.11
Post-SL	19.2 ± 4.0	20.3 ± 7.6	0.38
△SL	0.5 ± 4.3	3.8 ± 5.1	**0.02** ^*∗*^
Pre-SS	32.8 ± 9.9	33.2 ± 11.3	0.87
Post-SS	33.5 ± 7.1	35.0 ± 9.4	0.53
△SS	0.8 ± 10.7	1.8 ± 8.5	0.66
Pre-HOD	8.2 ± 2.2	7.9 ± 2.7	0.76
Post-HOD	10.1 ± 2.1	10.3 ± 2.8	0.72
△HOD	1.9 ± 2.0	2.4 ± 2.6	0.47
Pre-PI	49.7 ± 10.4	50.5 ± 10.4	0.76
Post-PI	50.0 ± 9.5	50.0 ± 8.8	0.97
△PI	0.3 ± 6.4	−0.6 ± 5.0	0.51
Pre-PT	17.9 ± 4.2	18.9 ± 4.7	0.42
Post-PT	20.1 ± 5.2	15.1 ± 5.4	**0.001** ^*∗*^
△PT	2.2 ± 4.2	−3.7 ± 5.3	**<0.001** ^*∗*^
Pre-NRS	7.5 ± 0.8	7.7 ± 1.0	0.34
Post-NRS	4.5 ± 0.7	2.8 ± 0.7	**<0.001** ^*∗*^
△NRS (%)	39.3 ± 7.3	64.0 ± 7.8	**<0.001** ^*∗*^
Pre-ODI	57.1 ± 5.8	54.6 ± 6.0	0.12
Post-ODI	29.9 ± 6.1	22.4 ± 6.1	**<0.001** ^*∗*^
△ODI (%)	47.3 ± 10.9	58.5 ± 11.9	**0.001** ^*∗*^

Abbreviations: LL, lumbar lordosis; SL, segmental lordosis; SS, sacral slope; HOD, height of intervertebral disc; PI, pelvic incidence; PT, pelvic tilt; PLBP, persistent low back pain; NRS, Numeric Rating Scale; ODI, Oswestry Disability Index. ^*∗*^*p* values in boldface indicate statistical significance (*p* < 0.05).

**Table 4:** Correlation analysis between sagittal balance parameters and clinical outcomes.

	△LL	△SL	△SS	△HOD	△PI	△PT	△NRS	△ODI
△LL		**0.37 (** **p<0** **.001** **)**	**0.52 (** **p<0** **.001** **)**	0.07 (*p*=0.47)	**0.23 (** **p=0** **.02** **)**	**−0.52 (** **p<0** **.001** **)**	0.09 (*p*=0.36)	0.15 (*p*=0.13)
△SL			**0.35 (** **p<0** **.001** **)**	0.17 (*p*=0.08)	0.01 (*p*=0.89)	**−0.33 (** **p<0** **.001** **)**	**0.27 (** **p=0** **.006** **)**	**0.41 (** **p<0** **.001** **)**
△SS				0.09 (*p*=0.35)	**0.33 (** **p=0** **.001** **)**	**−0.35 (** **p<0** **.001** **)**	0.05 (*p*=0.59)	0.12 (*p*=0.22)
△HOD					0.07 (*p*=0.49)	−0.04 (*p*=0.67)	0.12 (*p*=0.20)	0.08 (*p*=0.44)
△PI						0.04 (*p*=0.65)	0.04 (*p*=0.72)	−0.11 (*p*=0.26)
△PT							**−0.40 (** **p<0** **.001** **)**	**−0.22 (** **p=0** **.03** **)**
△NRS								**0.43 (** **p<0** **.001** **)**
△ODI								

Abbreviations: △LL, change of lumbar lordosis; △SL, change of segmental lordosis; △SS, change of sacral slope; △HOD, change of height of the intervertebral disc; △PI, change of pelvic incidence; △PT, change of pelvic tilt; △NRS, Numeric Rating Scale; △ODI, Oswestry Disability Index. ^*∗*^*p* values in boldface indicate statistical significance (*p* < 0.05).

**Table 5:** Logistic analysis of each variable for postoperative PLBP.

Variables	Partial correlation coefficient	Exp (B) (95% CI)	*p* value
Sex	0.32	1.38 (0.35, 5.50)	0.65
Age	0.004	1.00 (0.95, 1.06)	0.89
Operative levels	−0.26	0.77 (0.17, 3.47)	0.74
△LL	0.07	1.07 (0.98, 1.17)	0.12
△SL	−0.12	0.89 (0.76, 1.03)	0.11
△SS	0.05	1.05 (0.96, 1.14)	0.28
△HOD	−0.07	0.94 (0.73, 1.20)	0.60
△PI	−0.03	0.98 (0.87, 1.10)	0.68
△PT	0.32	1.38 (1.15, 1.65)	**0.001** ^*∗*^

Abbreviations: △LL, change of lumbar lordosis; △SL, change of segmental lordosis; △SS, change of sacral slope; △HOD, change of the height of intervertebral disc; △PI, change of pelvic incidence; △PT, change of pelvic tilt; PLBP, persistent low back pain. ^*∗*^*p* values in boldface indicate statistical significance (*p* < 0.05).

## Data Availability

No data were used to support this study.
